# Divergent trends of human immunodeficiency virus/acquired immunodeficiency syndrome (HIV/AIDS), syphilis, and gonorrhea in China: a national age-period-cohort analysis, 2006–2020

**DOI:** 10.3389/fpubh.2025.1699970

**Published:** 2025-12-12

**Authors:** Huihui Tuo, Fang Feng, Ronggui Tao, Zhongyu Wang, Shuo Feng, Luqian Zhang, Yan Zheng

**Affiliations:** 1 Department of Dermatology, The First Affiliated Hospital of Xi’an Jiaotong University, Xi’an, China; 2 Department of Gynecology and Obstetrics, The First Affiliated Hospital of Xi’an Jiaotong University, Xi’an, China; 3 Department of Urology, The First Affiliated Hospital of Xi’an Jiaotong University, Xi’an, China; 4 Shaanxi Provincial Centre for Disease Prevention and Control, Xi’an, China

**Keywords:** HIV/AIDS, syphilis, gonorrhea, reported incidence, joinpoint regression model, age-period-cohort model, China

## Abstract

**Objective:**

Sexually transmitted diseases (STDs) remain a significant public health challenge globally and in China. This study analyzes incidence trends of three nationally notifiable STDs to assess their disease burden.

**Methods:**

Based on national surveillance data (2006–2020) from the Chinese Center for Disease Control and Prevention, this study analyzed incidence trends of human immunodeficiency virus/acquired immunodeficiency syndrome (HIV/AIDS), syphilis, and gonorrhea using joinpoint regression. Age-period-cohort modeling was subsequently employed to evaluate the independent effects of age, period, and birth cohort. Rate ratios (RRs) with 95% confidence intervals (CIs) were calculated to quantify these associations.

**Results:**

Between 2006 and 2020, China observed a significant increase in the incidence of HIV/AIDS and syphilis (*p* < 0.001). Although gonorrhea incidence declined overall (*p* < 0.05), a notable increase was observed among adolescents under 20 years of age (*p* < 0.05). The average annual reported incidence was highest for syphilis (28.41 per 100,000), followed by gonorrhea (8.65 per 100,000) and HIV/AIDS (2.78 per 100,000). Geographically, HIV/AIDS incidence was highest in southwestern China. Syphilis incidence was elevated in the southwestern, southern, and northwestern regions, while gonorrhea showed a distinct concentration in southern China. Age-effect analysis revealed a bimodal pattern for both HIV/AIDS and syphilis, with incidence peaks in the 20–39 and ≥60 age groups. In contrast, gonorrhea showed a single peak in the 20–39 age group. Period-effect analysis, using the 2011–2015 period as reference (RR = 1), indicated evolving disease risks in subsequent years: HIV/AIDS risk increased markedly in 2016–2020 (RR = 1.53, 95% CI: 1.41–1.65), syphilis risk increased modestly (RR = 1.14, 95% CI: 1.06–1.22), while gonorrhea risk initially declined in 2006–2010 (RR = 1.43, 95% CI: 1.34–1.54) before a slight rise in 2016–2020 (RR = 1.11, 95% CI: 1.03–1.19). Cohort analysis showed that HIV/AIDS risk was highest among individuals born in 2011–2015 (RR = 124.81, 95% CI: 36.82–423.08). The peak risk for syphilis occurred in the 2006–2010 birth cohort (RR = 5.08, 95% CI: 1.28–19.99), while gonorrhea risk was highest in the 1921–1925 birth cohort (RR = 15.46, 95% CI: 2.00–119.62).

**Conclusion:**

HIV/AIDS and syphilis present growing threats in China, disproportionately affecting young adults and older populations, with increasing burden in recent birth cohorts. Although gonorrhea shows overall decline, its rise in adolescents warrants attention. A tiered prevention strategy is recommended: enhanced screening and education for HIV/syphilis in high-risk groups and regions, alongside sustained gonorrhea surveillance in key populations.

## 1 Introduction

Sexually transmitted diseases (STDs) represent a significant public health issue worldwide and in China (1). In response, China has classified three major STDs—human immunodeficiency virus/acquired immunodeficiency syndrome (HIV/AIDS), syphilis, and gonorrhea—as notifiable infectious diseases and implemented comprehensive monitoring and prevention systems.

HIV/AIDS, caused by *human immunodeficiency virus* (HIV), is a chronic infectious disease characterized by specific attacks on the immune system, leading to opportunistic infections and malignancies with high mortality. Globally, HIV incidence initially increased followed by a declining trend (2). In contrast, China has experienced a consistent annual rise in incidence, with the primary transmission route shifting from early blood-borne exposure to sexual contact (3, 4).

Syphilis, caused by *Treponema pallidum*, progresses through primary (chancre), secondary (syphilid), tertiary (visceral/neurologic involvement), and latent stages. Congenital syphilis may result in miscarriage, stillbirth, or congenital syphilis in newborns. Globally, syphilis prevalence shows distinct regional disparities, with high rates in sub-Saharan Africa, Southeast Asia, and South America (5). In China, reported syphilis incidence has shown a fluctuating yet rising trend, accompanied by a growing proportion of latent cases (6).

Gonorrhea, caused by *Neisseria gonorrhoeae*, mainly invades the genitourinary tract, presenting with symptoms such as dysuria and/or urethral purulent discharge. If untreated, it may lead to chronic reproductive inflammation, ectopic pregnancy, or infertility (7). Although global surveillance data indicate a declining trend in gonorrhea incidence (8), the decrease in China has been limited. Challenges such as asymptomatic transmission and emerging antimicrobial resistance further complicate clinical management and public health control (9, 10).

Overall, the reported incidence of notifiable STDs in China continues to rise steadily. However, current surveillance remains largely descriptive, lacking insight into the underlying drivers of these trends—a gap that impedes the development of targeted interventions. Therefore, this study employs joinpoint regression and age-period-cohort modeling to systematically analyze long-term trends in three major STDs and quantify the independent effects of age, period, and cohort. The results aim to inform the design of stratified, evidence-based prevention strategies.

## 2 Methods

### 2.1 Data collection

Reported case data for HIV/AIDS, syphilis, and gonorrhea across different age groups and geographic regions in China (2006–2020) were obtained from the Public Health Science Data Center of the Chinese Center for Disease Control and Prevention.^1^ Mainland China’s 31 provincial-level divisions were categorized into seven geographical regions (data for Hong Kong, Macao, and Taiwan were not included): Northeast (Liaoning, Jilin, Heilongjiang), North (Beijing, Tianjin, Hebei, Shanxi, Inner Mongolia), East (Shanghai, Jiangsu, Zhejiang, Anhui, Fujian, Jiangxi, Shandong), South (Guangdong, Guangxi, Hainan), Central (Henan, Hubei, Hunan), Northwest (Shaanxi, Gansu, Qinghai, Ningxia, Xinjiang), and Southwest (Chongqing, Sichuan, Guizhou, Yunnan, Tibet). Population data for corresponding years were sourced from the National Bureau of Statistics Yearbooks.^2^ Annual reported incidence rates for the three STDs were calculated for each geographical division.

### 2.2 Joinpoint regression analysis

Joinpoint regression is a segmented linear regression method for time-series trend analysis. It determines the optimal number of statistically significant inflection points within the study period through iterative computation, thereby dividing the overall trend into multiple linear segments with different slopes and modeling the incidence trend for each segment separately (11). The study population was stratified into four age groups: <20 years, 20–39 years, 40–59 years, and ≥60 years. Using Joinpoint software (version 4.9.1.0), we calculated the annual percent change (APC), average annual percent change (AAPC), and corresponding 95% confidence intervals (CIs) for the overall population and for each age stratum. An APC > 0 indicates an increasing trend, while APC < 0 indicates a decreasing trend; when APC = AAPC, it represents a monotonic trend.

### 2.3 Age-period-cohort analysis

The age-period-cohort analysis is a statistical approach used to explore the effects of age, time period, and birth cohort on disease incidence and to analyze long-term trends in disease development. The analysis was performed using the web-based age-period-cohort tool developed by the U.S. National Cancer Institute^3^ (12). This tool is based on the model assumption that age, period, and cohort effects are additive and that individuals within each group share homogeneous risks. To address the inherent identifiability problem due to the exact linear dependence among the three dimensions, the intrinsic estimator method was applied by imposing equality constraints on model parameters and focusing on estimable functions, such as the longitudinal age curve and period/cohort rate ratios. Data were categorized into 18 five-year age groups (including a combined group for ages ≥85 years), three five-year periods (2006–2010, 2011–2015, and 2016–2020), and 20 five-year birth cohorts (1921–1925 to 2016–2020). Rate ratios (RRs) and 95% CIs were estimated using the 2011–2015 period and the 1971–1975 birth cohort as reference groups.

## 3 Results

### 3.1 Overall trends in reported incidence of HIV/AIDS, syphilis, and gonorrhea in China

**Figure 1:**
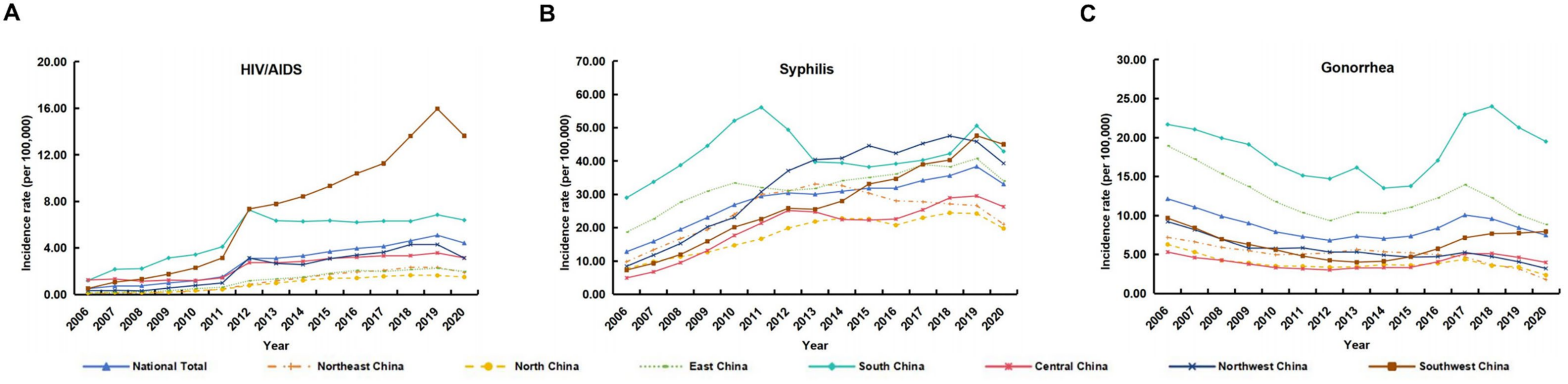
Annual reported incidence rates of human immunodeficiency virus/acquired immunodeficiency syndrome (HIV/AIDS) **(A)**, syphilis **(B)**, and gonorrhea **(C)** by region in China, 2006–2020.

Between 2006 and 2020, China reported 564,917 HIV/AIDS cases, corresponding to an average annual incidence of 2.78 per 100,000 population. The peak was observed in 2019, with 71,204 cases (5.10 per 100,000). Nationally, HIV/AIDS incidence showed a sustained upward trend, with the most pronounced increase and highest burden concentrated in Southwest China (Figure 1A).

Syphilis was the most frequently reported among the three diseases, with 5,769,128 reported cases and an average annual incidence of 28.41 per 100,000. The highest number of cases and incidence rate also occurred in 2019, totaling 535,819 cases (38.37 per 100,000). Syphilis rates rose across all regions, particularly in the southwest, south, and northwest (Figure 1B).

Gonorrhea accounted for 1,755,893 cases during this period, with an average annual incidence of 8.65 per 100,000. Incidence peaked in 2006 at 158,795 cases (12.14 per 100,000), and exhibited a fluctuating trend nationwide. The highest disease burden was observed in south China, followed by the eastern region (Figure 1C).

### 3.2 Joinpoint regression analysis of HIV/AIDS, syphilis, and gonorrhea incidence in China

**Figure 2:**
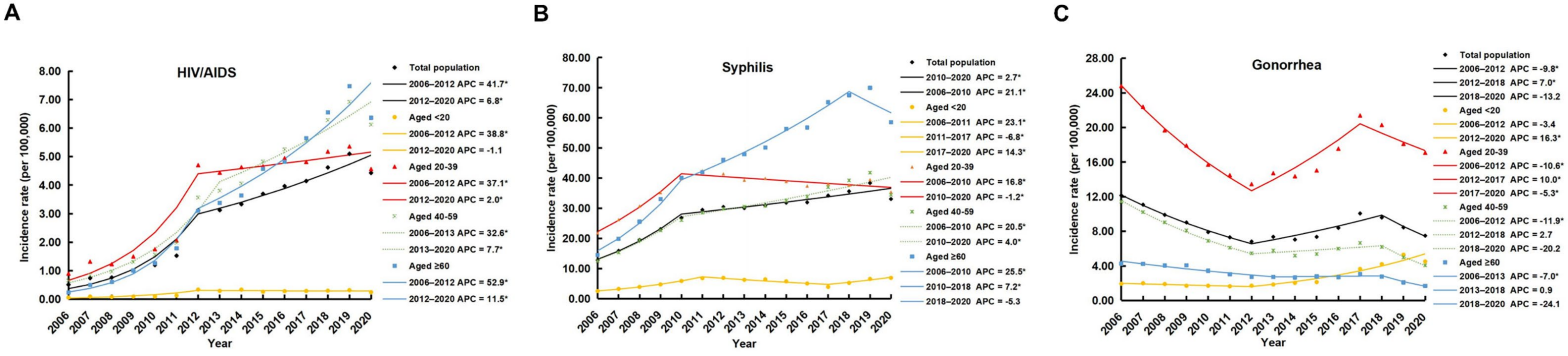
Joinpoint regression analysis of incidence trends for HIV/AIDS **(A)**, syphilis **(B)**, and gonorrhea **(C)** across age groups in China, 2006–2020. *Significant Annual Percent Change (APC) at *α* < 0.05.

Between 2006 and 2020, HIV/AIDS incidence in China showed a significant overall increase (AAPC = 20.5, *t* = 6.8, *p* < 0.001) (Figure 2A; Table 1). Age-stratified analyses consistently revealed rising trends across all age groups: <20 years (AAPC = 14.3, *t* = 4.7, *p* < 0.001), 20–39 years (AAPC = 15.8, *t* = 8.4, *p* < 0.001), 40–59 years (AAPC = 19.5, *t* = 6.7, *p* < 0.001), and ≥60 years (AAPC = 27.6, *t* = 3.6, *p* < 0.001).

**Table 1:** Joinpoint regression analysis of HIV/AIDS, syphilis, and gonorrhea incidence in China from 2006 to 2020.

Disease	Age group	Total interval			Trend 1				Trend 2				Trend 3			
		AAPC and 95% CI	*t*	*p*-value	Duration	APC and 95% CI	*t*	*p*-value	Duration	APC and 95% CI	*t*	*p*-value	Duration	APC and 95% CI	*t*	*p*-value
HIV/AIDS	Total population	20.5 (14.2, 27.2)	6.8	<0.001	2006–2012	41.7 (23.1, 63.1)	5.5	<0.001	2012–2020	6.8 (4.6, 9.0)	7.0	<0.001				
	Aged <20	14.3 (8.1, 21.0)	4.7	<0.001	2006–2012	38.8 (20.2, 60.2)	5.1	<0.001	2012–2020	−1.1 (−3.9, 1.8)	−0.8	0.418				
	Aged 20–39	15.8 (11.9, 19.8)	8.4	<0.001	2006–2012	37.1 (25.5, 49.6)	8.0	<0.001	2012–2020	2.0 (0.3, 3.8)	2.6	0.026				
	Aged 40–59	19.5 (13.4, 25.9)	6.7	<0.001	2006–2013	32.6 (18.2, 48.7)	5.5	<0.001	2013–2020	7.7 (4.5, 11.0)	5.5	<0.001				
	Aged ≥60	27.6 (11.8, 45.8)	3.6	<0.001	2006–2012	52.9 (7.8, 116.8)	2.7	0.022	2012–2020	11.5 (7.8, 15.2)	7.3	<0.001				
Syphilis	Total population	7.6 (5.2, 10.2)	6.2	<0.001	2006–2010	21.1 (10.9, 32.1)	4.9	0.001	2010–2020	2.7 (1.5, 3.9)	5.2	<0.001				
	Aged <20	7.5 (5.1, 10.0)	6.3	<0.001	2006–2011	23.1 (17.0, 29.4)	9.7	<0.001	2011–2017	−6.8 (−9.8, −3.8)	−5.2	0.001	2017–2020	14.3 (6.4, 22.8)	4.4	0.003
	Aged 20–39	3.7 (2.6, 4.8)	6.5	<0.001	2006–2010	16.8 (12.3, 21.6)	8.8	<0.001	2010–2020	−1.2 (−1.8, −0.5)	−3.8	0.004				
	Aged 40–59	8.5 (4.8, 12.3)	4.6	<0.001	2006–2010	20.5 (5.7, 37.4)	3.2	0.010	2010–2020	4.0 (2.3, 5.7)	5.4	<0.001				
	Aged ≥60	10.2 (8.0, 12.3)	9.7	<0.001	2006–2010	25.5 (17.1, 34.5)	7.7	<0.001	2010–2018	7.2 (5.9, 8.5)	13.5	<0.001	2018–2020	−5.3 (−12.0, 2.0)	−1.7	0.128
Gonorrhea	Total population	−3.5 (−6.1, −0.8)	−2.5	0.012	2006–2012	−9.8 (−12.2, −7.4)	−9.1	<0.001	2012–2018	7.0 (2.9, 11.3)	4.1	0.005	2018–2020	−13.2 (−28.0, 4.5)	−1.8	0.114
	Aged <20	7.4 (2.3, 12.8)	2.9	0.004	2006–2012	−3.4 (−13.8, 8.2)	−0.7	0.509	2012–2020	16.3 (11.1, 21.8)	7.3	<0.001				
	Aged 20–39	−2.5 (−4.1, −1.0)	−3.2	0.001	2006–2012	−10.6 (−12.1, −9.0)	−15.4	<0.001	2012–2017	10.0 (5.9, 14.2)	5.9	0.001	2017–2020	−5.3 (−10.1, −0.3)	−2.5	0.040
	Aged 40–59	−7.3 (−10.2, −4.2)	−4.5	<0.001	2006–2012	−11.9 (−13.8, −10.0)	−13.8	<0.001	2012–2018	2.7 (−1.2, 6.8)	1.6	0.152	2018–2020	−20.2 (−37.2, 1.3)	−2.2	0.060
	Aged ≥60	−7.0 (−11.9, −1.8)	−2.6	0.009	2006–2013	−7.0 (−9.6, −4.3)	−6.0	0.001	2013–2018	0.9 (−7.1, 9.6)	0.3	0.807	2018–2020	−24.1 (−49.1, 13.2)	−1.6	0.147

HIV/AIDS, human immunodeficiency virus/acquired immunodeficiency syndrome; AAPC, average annual percent change; APC, annual percent change; 95% CI, 95% confidence interval.

Similarly, the overall incidence of syphilis increased significantly (AAPC = 7.6, *t* = 6.2, *p* < 0.001), with rising trends observed in all age groups: <20 years (AAPC = 7.5, *t* = 6.3, *p* < 0.001), 20–39 years (AAPC = 3.7, *t* = 6.5, *p* < 0.001), 40–59 years (AAPC = 8.5, *t* = 4.6, *p* < 0.001), and ≥60 years (AAPC = 10.2, *t* = 9.7, *p* < 0.001) (Figure 2B; Table 1).

In contrast, gonorrhea incidence exhibited an overall decline during this period (AAPC = −3.5, *t* = −2.5, *p* < 0.05) (Figure 2C; Table 1). Age-specific analyses showed a significant increase among individuals under 20 years (AAPC = 7.4, *t* = 2.9, *p* < 0.05), while significant decreases were observed in the 20–39 years (AAPC = −2.5, *t* = −3.2, *p* < 0.05), 40–59 years (AAPC = −7.3, *t* = −4.5, *p* < 0.001), and ≥60 years groups (AAPC = −7.0, *t* = −2.6, *p* < 0.05).

### 3.3 Effects of age, period, and cohort on the incidence of HIV/AIDS, syphilis, and gonorrhea in China

#### 3.3.1 Age effect of reported HIV/AIDS, syphilis, and gonorrhea incidence

**Figure 3:**
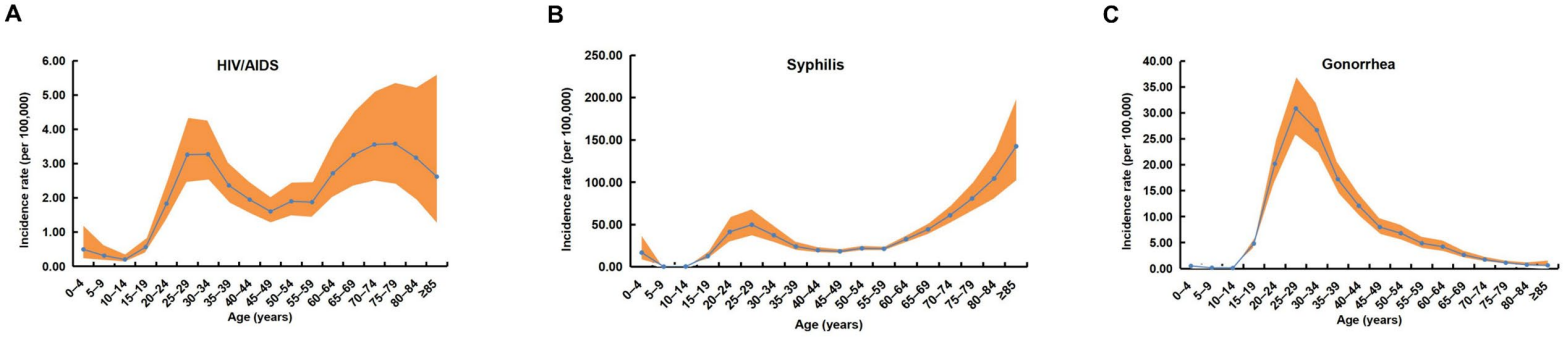
Age effects on the incidence of three notifiable sexually transmitted diseases (STDs) in China, 2006–2020. **(A)** Age-specific incidence curve with 95% confidence intervals (CIs) for HIV/AIDS. **(B)** Age-specific incidence curve with 95% CIs for syphilis. **(C)** Age-specific incidence curve with 95% CIs for gonorrhea. The orange-red shaded areas represent the 95% CIs.

After adjusting for period and cohort effects, the reported incidence of HIV/AIDS from 2006 to 2020 exhibited dynamic fluctuations across age groups, characterized by a bimodal distribution with two distinct peaks and troughs (Figure 3A). The troughs were observed in the 0–19 and 40–59 year age groups, with the lowest incidence occurring in the 10–14 year group (0.20 per 100,000). The peaks were seen in the 20–39 and ≥60 year groups, with the highest incidence in the 75–79 year group (3.58 per 100,000).

Similarly, the incidence of syphilis displayed dynamic age-related fluctuations, resembling the pattern observed for HIV/AIDS. Troughs were noted in the 0–19 and 40–59 year groups, with the lowest rate in the 10–14 year group (0.46 per 100,000) (Figure 3B). Peaks occurred in the 20–39 and ≥60 year groups, with the highest incidence observed in the ≥85 years group (142.46 per 100,000).

In contrast, gonorrhea incidence exhibited a unimodal distribution, increasing initially before declining with advancing age (Figure 3C). The peak incidence was observed in the 20–39 year group, with the highest rate in the 25–29 year subgroup (30.87 per 100,000). The lowest incidence occurred in the 10–14 year group (0.11 per 100,000).

#### 3.3.2 Period effect of reported HIV/AIDS, syphilis, and gonorrhea incidence

**Figure 4:**
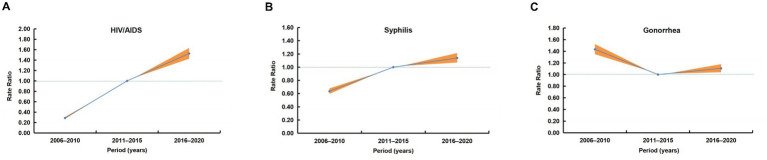
Period effects on the incidence of three notifiable STDs in China, 2006–2020. **(A)** Overall period rate ratios with 95% CIs for HIV/AIDS. **(B)** Period rate ratios with 95% CIs for syphilis. **(C)** Period rate ratios with 95% CIs for gonorrhea. The 2011–2015 period serves as the reference (rate ratio = 1). The orange-red shaded areas represent the 95% CIs.

After adjusting for age and cohort effects, the period effect on HIV/AIDS incidence from 2006 to 2020 showed a significant upward trend (Figure 4A). Using the 2011–2015 period as the reference (RR = 1), the rate ratio was 0.29 (95% CI, 0.26–0.32) during 2006–2010 and increased to 1.53 (95% CI, 1.41–1.65) in 2016–2020.

Similarly, a rising period effect was observed for syphilis incidence (Figure 4B). With the 2011–2015 period as reference (RR = 1), the rate ratio was 0.64 (95% CI, 0.59–0.69) in 2006–2010 and rose to 1.14 (95% CI, 1.06–1.22) during 2016–2020.

In contrast, gonorrhea incidence exhibited a declining-then-rising period effect (Figure 4C). Compared with the 2011–2015 reference period (RR = 1), the rate ratio was 1.43 (95% CI, 1.34–1.54) in 2006–2010 and decreased to 1.11 (95% CI, 1.03–1.19) in 2016–2020.

#### 3.3.3 Cohort effect of reported HIV/AIDS, syphilis, and gonorrhea incidence

**Figure 5:**
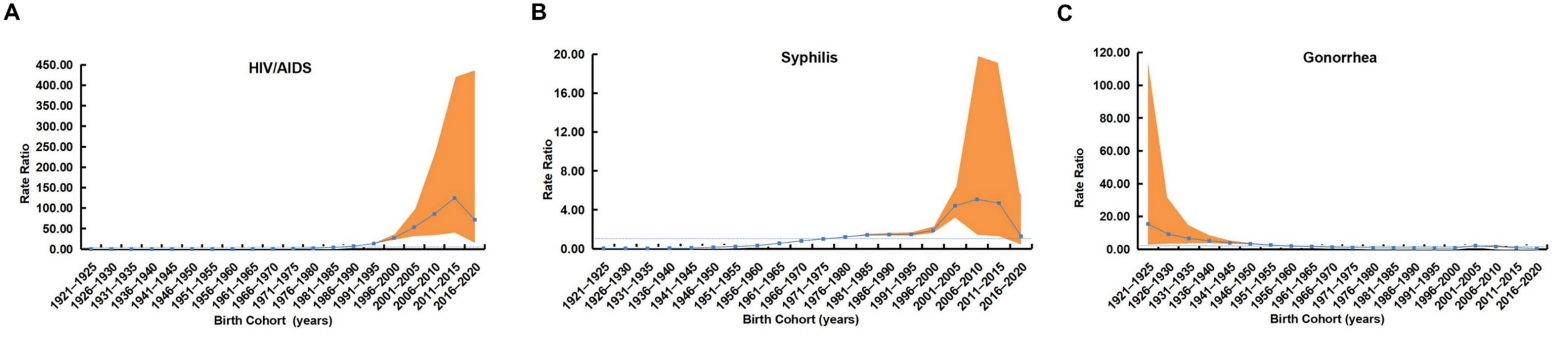
Cohort effects on the incidence of three notifiable STDs in China, 2006–2020. **(A)** Overall cohort rate ratios with 95% CIs for HIV/AIDS. **(B)** Cohort rate ratios with 95% CIs for syphilis. **(C)** Cohort rate ratios with 95% CIs for gonorrhea. The 1971–1975 birth cohort serves as the reference (rate ratio = 1). The orange-red shaded areas represent the 95% CIs.

After adjusting for age and period effects, the cohort effect for HIV/AIDS incidence from 2006 to 2020 showed an initial increasing trend followed by a recent decline (Figure 5A). Using the 1971–1975 birth cohort as reference (RR = 1), cohorts born between 1921 and 1970 exhibited rate ratio below 1, while those born from 1976 to 2020 showed rate ratios exceeding 1, with the peak risk observed in the 2011–2015 cohort (RR = 124.81, 95% CI: 36.82–423.08).

For syphilis incidence, the cohort effect similarly demonstrated an initial rise followed by a recent decline (Figure 5B). With the 1971–1975 cohort as reference (RR = 1), the rate ratio generally increased among cohorts born from 1921 to 2005, reaching its peak in the 2006–2010 cohort (RR = 5.08, 95% CI: 1.28–19.99), followed by a slight decline in the 2011–2020 cohorts.

In contrast, gonorrhea incidence exhibited a distinct cohort pattern characterized by an initial decline followed by dynamic fluctuations (Figure 5C). Referenced to the 1971–1975 cohort (RR = 1), the highest risk was seen in the 1921–1925 cohort (RR = 15.46, 95% CI: 2.00–119.62). Rate ratios remained above 1 but showed a declining trend among cohorts born from 1921 to 1970, fell below 1 for the 1971–2000 and 2011–2020 cohorts, and rose again above 1 in the 2001–2010 cohorts.

## 4 Discussion

This study reveals distinct epidemiological patterns for major STDs in China during 2006–2020. The sustained increase in HIV and syphilis incidence across all age groups in China contrasts with declining global HIV trends (2), underscoring the distinctive nature of China’s evolving epidemic. Multiple interconnected factors contribute to the rising rates of HIV and syphilis. Policy initiatives, particularly the 2006 AIDS Prevention Regulations and subsequent five-year action plans, have enhanced disease surveillance and case detection (13–15). These policy measures coincided with significant behavioral changes, as evidenced by the shift in HIV transmission routes—sexual contact accounted for 96% of new infections in 2017, compared to 11% in 2005, including an increase in transmission among men who have sex with men to 26% (13). Social transformations have further influenced disease spread. Urbanization, internal migration, and social media use have collectively fostered more complex sexual networks (4, 16). By the end of 2014, China had approximately 253 million internal migrants, representing 18.5% of the total population (17), with this mobile population exhibiting higher rates of multiple sexual partnerships (18). Additionally, HIV-syphilis co-infection generates synergistic effects that accelerate transmission dynamics (3, 19, 20). In contrast, gonorrhea incidence showed overall decline, aligning with global trends (8). This reduction can be largely attributed to widespread condom promotion, the implementation of nucleic acid amplification tests (NAATs), and standardized treatment protocols (21). However, the rising incidence among adolescents and young adults underscores persistent gaps in sexual health education (22). Supporting this observation, Wang et al. reported a marked increase in gonorrhea incidence among Chinese males aged 15–19 after 2010, with an annual growth rate of 18.5% (15). This trend may be partially explained by earlier initiation of unprotected sexual activity among youth (17), highlighting the need for targeted prevention strategies for this vulnerable demographic.

The divergent efficacy of public health interventions against these three STDs appears to be shaped by their distinct biological characteristics. *Neisseria gonorrhoeae* appears to exhibit high transmissibility and relatively short incubation, characteristics that could enhance responsiveness to condom use and antibiotic therapy (23). In comparison, HIV infection tends to involve prolonged infectivity and a diagnostic window period, factors that might contribute to the need for broader intervention coverage (24, 25). *Treponema pallidum* infections can progress to latent stages lasting several years and show complex serological patterns, possibly contributing to persistent transmission risks in some cases (26).

Geographically, HIV/AIDS incidence remained highest in southwestern China, where initial outbreaks linked to drug trafficking routes established early footholds. Persistent socioeconomic disadvantages in the region further sustained transmission risks (27, 28). Syphilis showed concentrated incidence in southwestern, southern, and northwestern China, overlapping with HIV/AIDS distribution and suggesting synergistic spread (3). Gonorrhea was most prevalent in southern China, with secondary concentration in eastern regions. Higher population mobility and expanded commercial sex networks in these economically active areas facilitated both transmission and case detection (29, 30). These patterns reflect regional disparities in health literacy combined with effects of large-scale internal migration (21).

Age-period-cohort analysis identifies middle-aged and older adults as high-risk groups for HIV/AIDS and syphilis, while gonorrhea risk is concentrated in younger and middle-aged populations. Middle-aged and young adults face increased exposure due to higher sexual activity and more permissive attitudes. Current prevention programs focus predominantly on young people, neglecting the growing epidemic among older population. In Sichuan Province, China, reported HIV incidence in older adults rose from 0.001% in 2008 to 0.077% in 2019 (31). Extended lifespan due to antiretroviral therapy, combined with persistent sexual activity and low protection awareness in this group, contributes to this trend (2, 32). In contrast, gonorrhea’s distinct clinical presentation—with obvious symptoms and a short incubation—does not typically lead to chronic concealed transmission, which makes it more controllable through intervention. With China’s rapidly aging population (33), tailored strategies for older adults are imperative, including targeted education for seniors and families, expanded routine screening, and accessible, confidential testing services.

HIV/AIDS and syphilis show consistent upward period effects, while gonorrhea displays an initial decline followed by a recent increase. China’s expanded screening and targeted testing have improved detection of existing infections (2, 24). Interventions including pre-exposure prophylaxis expansion and reduced injection drug use have slowed STD growth (34), though challenges persist from asymptomatic transmission and drug resistance (24). The initial decline in gonorrhea incidence reflected improvements in public awareness and diagnostic techniques (21). However, the recent resurgence appears to be associated with multiple factors, including the increased detection sensitivity of NAATs, the growing prevalence of antimicrobial resistance in *Neisseria gonorrhoeae* (10, 35), and enhanced transmission opportunities facilitated by social media platforms and international travel (36). China recommended NAATs for gonorrhea diagnosis in 2007. However, uptake was low; a 2015 survey of 90 hospitals across 11 provinces showed that only 17.78% utilized this method (15). Establishing national resistance surveillance, expanding drug sensitivity testing, accelerating antibiotic development, and integrating risk alerts into social platforms are urgently needed.

The cohort effects for HIV/AIDS and syphilis show an initial increase followed by a decline, while gonorrhea demonstrates an initial decrease succeeded by dynamic fluctuations. Since the first identification of AIDS in China in 1985, the epidemic spread rapidly over the following decade, primarily through drug use and blood transfusion (37, 38). Subsequent shifts in social structure, sexual attitudes, and increased population mobility further accelerated the dissemination of sexually transmitted infections (4). The significant overlap in transmission routes between HIV and syphilis has resulted in largely synchronized cohort trends for both diseases (19). Although methodological limitations may partially overestimate the relative risk in recent birth cohorts, the analysis clearly indicates that newer generations bear a substantially higher disease burden compared to historical levels. In contrast, gonorrhea transmission patterns are more influenced by intergenerational behavioral differences. Widespread sexual health education has led to significantly increased condom use among younger populations, and enhanced screening among high-risk groups has effectively reduced transmission (23). However, cohort analysis further reveals a concerning trend of renewed pressure on gonorrhea incidence in later birth cohorts. These patterns highlight the differential evolution of STDs and underscore the need for tailored prevention strategies.

This study has several limitations. First, the analysis relied on aggregated national surveillance data for notifiable STDs from 2006 to 2020. While these data provide coverage across provinces and age groups, the absence of individual-level clinical records and stratified information by sex or geographic setting (urban vs. rural) limited more granular risk stratification. Second, the observation period ended in 2020 and thus did not capture potential disruptions caused by the Corona Virus Disease 2019 pandemic to STD prevention services, healthcare-seeking behaviors, or transmission patterns. Continued surveillance is needed to systematically evaluate these effects. Third, underreporting remains a concern, particularly in underserved and economically disadvantaged regions, which may have biased incidence trends. Fourth, the observed rise in incidence may reflect both true epidemiological increases and improvements in diagnostic sensitivity and reporting systems. Although the age-period-cohort model can distinguish effects among age, period, and cohort dimensions, it cannot eliminate systematic bias introduced by the evolution of surveillance systems. Moreover, the model’s inherent identifiability problem further complicates the interpretation of these three interrelated effects. Thus, causal inferences should be drawn cautiously.

## 5 Conclusion

HIV/AIDS and syphilis present growing public health threats in China, disproportionately affecting young/middle-aged adults and older populations, with increasing disease burden observed in recent birth cohorts. While gonorrhea shows an overall moderated threat, its recent resurgence among adolescent populations necessitates continued vigilance. Geographically, HIV/AIDS was most prevalent in southwestern China, syphilis in southwestern, southern, and northwestern regions, and gonorrhea in southern and eastern China. A tiered prevention strategy is recommended: in high HIV/syphilis prevalence areas, prioritized screening and targeted health education for key adult populations should be strengthened; in gonorrhea-concentrated regions, sensitive surveillance systems and enhanced adolescent sexual health education should be maintained to consolidate current control achievements and prevent resurgence.

## Data Availability

The original contributions presented in the study are included in the article/Supplementary material, further inquiries can be directed to the corresponding authors.

## Footnotes

1
https://www.phsciencedata.cn/Share/

2
https://www.stats.gov.cn/sj/ndsj/

3
https://analysistools.cancer.gov/apc/
