# Supplementary figures and images for: Divergent trends of human immunodeficiency virus/acquired immunodeficiency syndrome (HIV/AIDS), syphilis, and gonorrhea in China: a national age-period-cohort analysis, 2006–2020
Source: Front Public Health. 2025 Dec 12;13:1699970. doi: 10.3389/fpubh.2025.1699970 (PMC12741117; doi:10.3389/fpubh.2025.1699970)

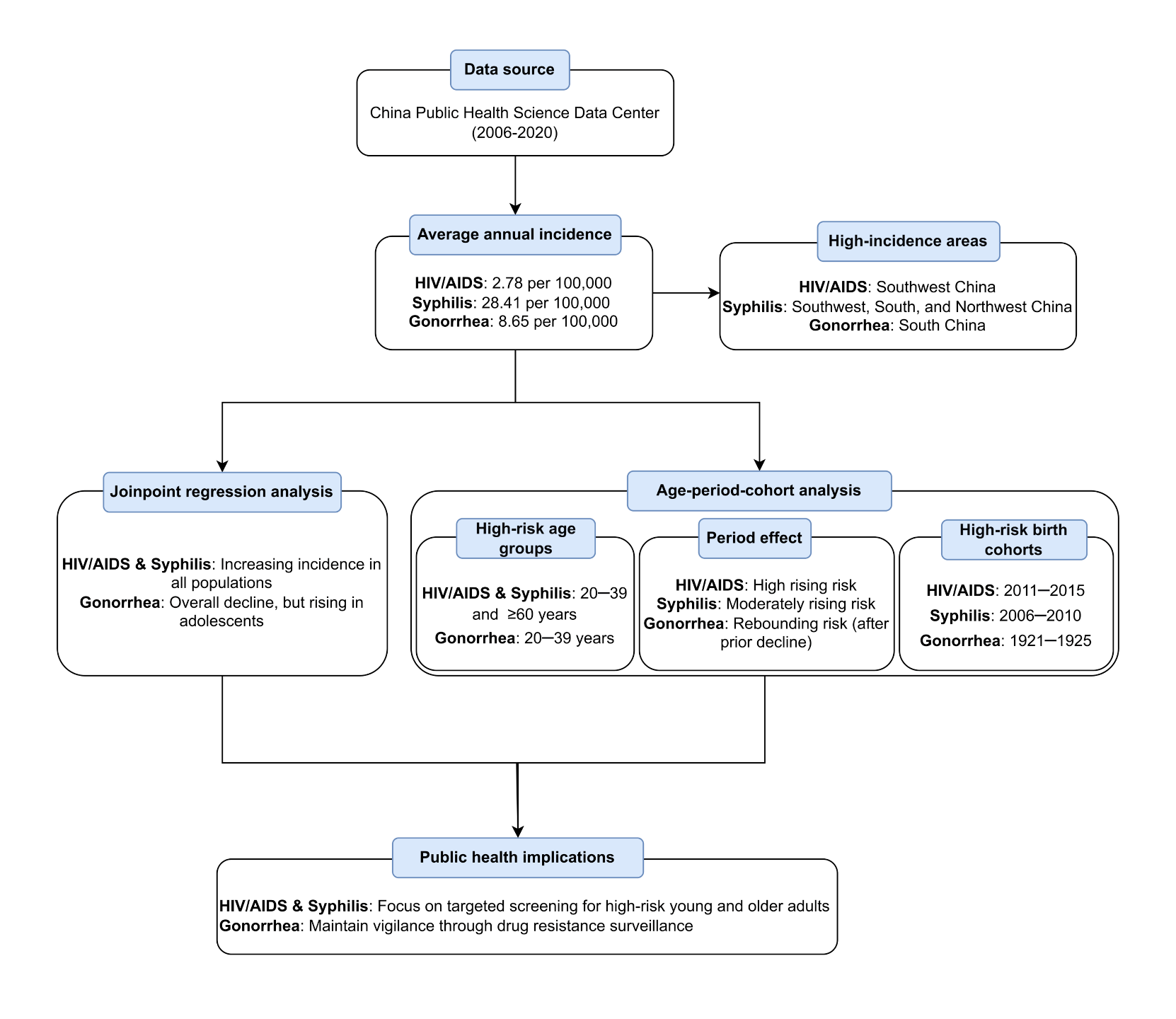

Supplement: Supplementary file 1 [file Image_1.TIFF]
